# Glucocorticoids Induce Nondipping Blood Pressure by Activating the Thiazide-Sensitive Cotransporter

**DOI:** 10.1161/HYPERTENSIONAHA.115.06977

**Published:** 2016-04-13

**Authors:** Jessica R. Ivy, Wilna Oosthuyzen, Theresa S. Peltz, Amelia R. Howarth, Robert W. Hunter, Neeraj Dhaun, Emad A.S. Al-Dujaili, David J. Webb, James W. Dear, Peter W. Flatman, Matthew A. Bailey

**Affiliations:** From the The British Heart Foundation Centre for Cardiovascular Science (J.R.I., W.O., T.S.P., A.R.H., R.W.H., N.D., D.J.W., J.W.D., M.A.B.) and The Centre for Integrative Physiology (P.W.F.), The University of Edinburgh, Edinburgh, United Kingdom; and Dietetics, Nutrition, and Biological Sciences Department, Queen Margaret University, Musselburgh, United Kingdom (E.A.S.A.-D.).

**Keywords:** cardiovascular disease, circadian rhythm, diabetes mellitus, hypertension, risk factors

## Abstract

Blood pressure (BP) normally dips during sleep, and nondipping increases cardiovascular risk. Hydrochlorothiazide restores the dipping BP profile in nondipping patients, suggesting that the NaCl cotransporter, NCC, is an important determinant of daily BP variation. NCC activity in cells is regulated by the circadian transcription factor per1. In vivo, circadian genes are entrained via the hypothalamic–pituitary–adrenal axis. Here, we test whether abnormalities in the day:night variation of circulating glucocorticoid influence NCC activity and BP control. C57BL6/J mice were culled at the peak (1:00 AM) and trough (1:00 PM) of BP. We found no day:night variation in NCC mRNA or protein but NCC phosphorylation on threonine^53^ (pNCC), required for NCC activation, was higher when mice were awake, as was excretion of NCC in urinary exosomes. Peak NCC activity correlated with peak expression of per2 and bmal1 (clock genes) and sgk1 and tsc22d3 (glucocorticoid-responsive kinases). Adrenalectomy reduced NCC abundance and blunted the daily variation in pNCC levels without affecting variation in clock gene transcription. Chronic corticosterone infusion increased bmal1, per1, sgk1, and tsc22d3 expression during the inactive phase. Inactive phase pNCC was also elevated by corticosterone, and a nondipping BP profile was induced. Hydrochlorothiazide restored rhythmicity of BP in corticosterone-treated mice without affecting BP in controls. Glucocorticoids influence the day:night variation in NCC activity via kinases that control phosphorylation. Abnormal glucocorticoid rhythms impair NCC and induce nondipping. Night-time dosing of thiazides may be particularly beneficial in patients with modest glucocorticoid excess.

## Abstract 2

Supplemental Digital Content is available in the text.

Blood pressure (BP) displays daily variation in healthy people, peaking midmorning and falling during night-time sleep.^1^ Loss or attenuation of the nocturnal BP dip (referred to as nondipping) incurs significant health risk by promoting cardiac, renal, and vascular injury.^2^ Nondipping is an early characteristic of diabetes mellitus preceding and predicting the development of microalbuminuria.^3^ In patients with chronic kidney disease, nocturnal hypertension is associated with a faster rate of decline in renal function.^4,5^ Even if clinic or mean 24-hour ambulatory BP is normal, loss of dipping significantly increases the risk of dialysis or incident cardiovascular mortality.^6,7^ Lowering sleep BP alone reduces risk of cardiovascular disease,^8^ suggesting that restoration of normal day:night BP variation is an important therapeutic goal.

Clinical studies show that the day:night variation in BP is strongly correlated with renal sodium excretion.^9^ The kidney is an important long-term regulator of BP and impaired pressure natriuresis may contribute to nocturnal hypertension^10^: BP remains elevated during sleep to facilitate sodium excretion and maintain 24-hour sodium balance.^11,12^ Certainly, hypertensive and prehypertensive patients have a blunted day:night pattern of sodium excretion and excrete a greater fraction of their daily output at night.^13^ Moreover, dietary sodium restriction can restore a dipping BP profile in salt-sensitive hypertensive patients,^14^ presumably by reducing sodium load below the day-time excretory threshold. Mechanistically, hydrochlorothiazide, either alone^15^ or in combination with amlodipine^16^ or valsartan,^17^ reduces night-time BP and restores dipping BP in nondipping hypertensive patients. This suggests that inappropriate activation of the thiazide-sensitive NaCl cotransporter (NCC) is a strong determinant of nocturnal BP.

NCC activity is modulated by multiple hormonal systems acting through a regulatory cascade of serine/threonine kinases, including WNKs, SPAK, and OSR1.^18^ These control phosphorylation at conserved residues in the N terminus, and thereby influence NCC activity and trafficking into the apical membrane.^18^ The distal tubule also expresses the canonical circadian transcription factors per1/2, bmal1, clock, and cry1/2,^19^ and a recent cell-line study demonstrated transcriptional regulation of NCC and WNK kinases by per1.^20^ It is increasingly evident that circadian transcription factors influence renal function.^21^ Glucocorticoids play an important role in the entrainment of renal clocks to the day:night cycle.^22^ In the aldosterone-sensitive distal nephron, glucocorticoid actions are normally limited because of metabolism by 11β-hydroxysteroid dehydrogenase type 2 (11βHSD2).^23^ However, modest glucocorticoid excess promotes sodium retention by activating transport in the aldosterone-sensitive distal nephron.^24^ Such perturbations, whether iatrogenic^25^ or because of conditions such as Cushing syndrome,^26^ also impair BP rhythmicity and induce a nondipping BP profile.

Because 11βHSD2 is only expressed in some cells of the distal convoluted tubule (DCT), we hypothesized that glucocorticoids set the day:night variation of NCC activity. Using the complementary approaches of adrenalectomy and chronic corticosterone infusion, we found that flattening the day:night variation in hypothalamic–pituitary–adrenal axis activity also flattened the variation in NCC activity, inducing a nondipping BP, which could be restored by thiazide therapy.

## Methods

Detailed Methods are available in the online-only Data Supplement.

### Animals

C57BL6J/Ola mice (Harlan, United Kingdom) were used between 2 and 4 months of age. Mice were acclimatized to a 12-hour light:dark cycle for at least 2 weeks before experiments and given free access to water and standard chow (maintenance diet 1; Special Diet Services, Essex, United Kingdom). The lights were turned on at 7:00 AM local time, and this was defined as Zeitgeber time 0 (ZT0); lights were turned off at 7 PM (ZT12). Experiments were performed under license from the UK Home Office and after approval by the University veterinary services.

### Kidney Collection

Mice were culled by cervical dislocation <1-minute after removal from the holding room. Culls were performed at both 1:00 PM (ZT6) and 1:00 AM (ZT18) local time. The kidneys were rapidly excised and decapsulated, snap-frozen on dry ice and stored at −80°C.

### Bilateral Adrenalectomy

This was performed under isoflurane anesthesia and buprenorphine (Vetergesic 0.1 mg/kg sc) analgesia. After recovery, adrenalectomized and control mice were individually housed and given ad libitum access to 0.9% saline and tap water.

### Chronic Corticosterone Infusion

Elastomer pellets were used to encapsulate corticosterone for slow release. In vitro, the release rate was ≈3.7 mg/kg per day, and this was maintained during a 4-week period. Pellets were implanted subcutaneously under isoflurane anesthesia and buprenorphine analgesia (Vetergesic 0.1 mg/kg sc).

### Plasma Corticosterone Measurements

Approximately 20-μL blood was sampled from conscious mice via the tail vein at local time 7:00 AM and 7:00 PM, unless otherwise stated. Plasma was stored at −80°C before measurement of corticosterone concentration by commercial EIA (Enzo Life Sciences, Exeter, United Kingdom).

### Daily Variation in Renal Excretion

Control mice were single housed in metabolic cages, and after 5 days of acclimatization, food intake, water intake, urine output, and body weight were measured at 12-hour intervals. The concentration of sodium in urine was measured by flame photometry and used to calculate excretion rate in μmol/h. Urine concentration of both aldosterone and corticosterone was measured by ELISA.^27^

### Measurement of NCC in Urinary Exosomes

The number of exosomes in urine samples was measured by Nanoparticle Tracking Analysis (NanoSight Ltd, Amesbury, United Kingdom), as described.^28^

### Immunoblotting

Western blots were performed on homogenized whole-kidney samples as we have described,^29^ using primary antibodies against NCC, pThr53-NCC, anti-pThr58-NCC, anti-pSer71-NCC, and anti-NKCC2. Images were developed by electrochemiluminescence and quantified by densitometry using ImageJ, as described.^30^ The cross-reactivity of NCC phospho-antibodies was determined by immunoprecipitation experiments. Anti-pThr53-NCC and anti-pSer71-NCC were selective for NCC but anti-pThr58-NCC showed significant cross-reactivity with NKCC2 (Figure S1).

### Quantitative Polymerase Chain Reaction

RNA was extracted from whole kidney and used for quantitative polymerase chain reactions with the Roche Universal ProbeLibrary (online-only Data Supplement; Table). TBP, HPRT, and 18S rRNAs were used as endogenous control genes.

**Table.**
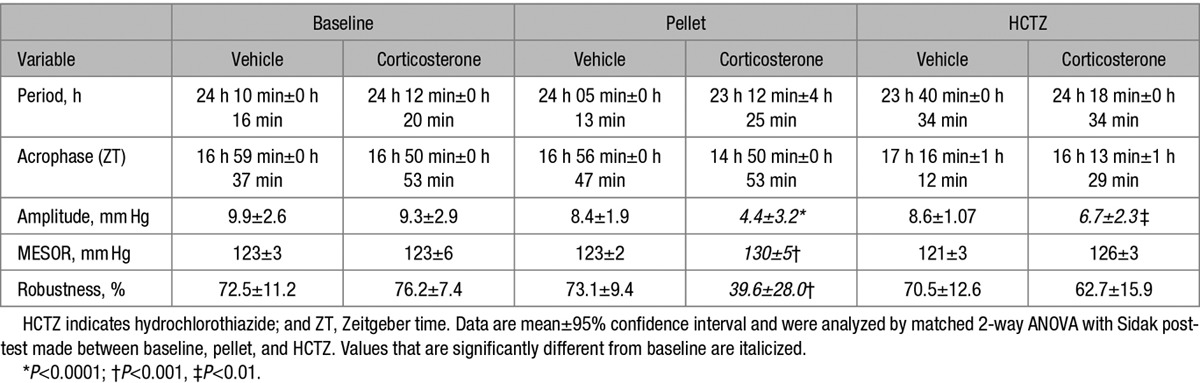
Cosinor Analysis of Systolic Blood Pressure in C57BL6 Mice Treated With Chronic Corticosterone or Vehicle Followed by Chronic Hydrochlorothiazide Treatment (80 mg/kg)

### Immunofluorescence

Kidneys were fixed by aortic perfusion of 4% paraformaldehyde and then embedded in paraffin, sectioned at 5 μm and mounted onto glass slides. Sections were double-immunostained to colocalize NCC expression with glucocorticoid receptor, mineralocorticoid receptor (MR), or 11βHSD2.

### BP Measurement

Radiotelemetry devices were inserted into male C57BL/6 mice under isoflurane anesthesia. Data were collected during a 1-minute period every 30 minutes at an acquisition rate of 1 kHz. Basal measurements were obtained >7 days before corticosterone (n=5) or blank (n=6) elastomer pellets were implanted subcutaneously. After 11 days, hydrochlorothiazide (80 mg/kg per day) was administered in drinking water. Hydrochlorothiazide concentration in plasma samples taken on the final experimental day was measured by liquid chromatography–mass spectrometry.

### Statistical Analysis

Data are presented as mean±95% confidence interval. The number of biological replicates (n) for each experimental group is given in the figure legends along with the test used for statistical analysis. The arithmetic mean BP was calculated for periods of subjective day, when mice were asleep, and subjective night, when mice were awake. Cosinor analysis was also used to calculate mesor (rhythm-adjusted mean) as the central tendency, period, amplitude, and acrophase. Data were smoothed using a moving average during 5 hours. For each experimental phase (baseline, corticosterone, and thiazide), the smoothed data for each individual mouse was collected in bins of 5 consecutive days and fitted by the least squares methods to a cosinor curve using software available at www.circadian.org, as described.^31^ Robustness/prominence of the rhythm was calculated as the percentage of the variance accounted for by the cosinor model. Statistical significance of the goodness of fit was *P*<0.000001 for each analysis and was corrected for multiple tests.

## Results

### Basal Day:Night Variation in NCC Phosphorylation

Under basal conditions, BP and heart rate had a robust 24-hour rhythm, dipping during subjective day when mice were in the inactive phase (Figure S2). Urinary excretion of Na and K also had this variation, as did the urinary excretion of aldosterone and corticosterone (Figure S3).

**Figure 1.**
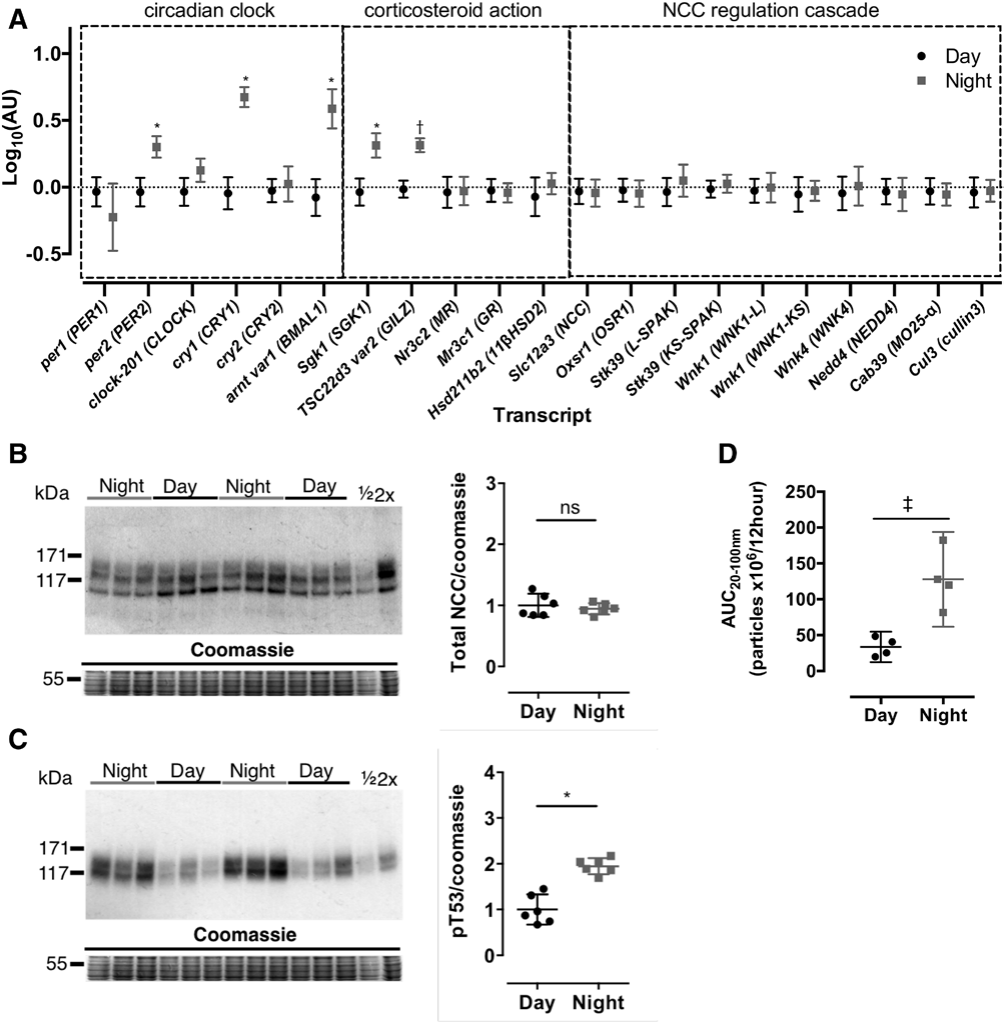
Day:night variation of gene expression (**A**), total NaCl cotransporter (NCC) protein (**B**), phosphorylated NCC (pT53-NCC; **C**), and exosomal NCC (**D**) in C57BL6 mice. Kidneys were harvested at Zeitgeber time (ZT) 6 (day) and ZT18 (night) for immunoblots and quantitative polymerase chain reaction using whole kidney homogenate. **A**–**C**, Exosomal NCC was measured by Nano-tracking gated for 20- to 100-nm particles labeled with Q-dot conjugated NCC antibody in 12-hour urine samples collected between ZT0–12 (day/sleep) and ZT12–0 (night/active). Two-way ANOVA with post hoc Sidak correction was carried out on gene expression data (n=15). Day:night total (n=6) and phosphorylated NCC (n=6) normalized densities in arbitrary units were compared by unpaired *t* tests. Exosomal NCC was corrected for urinary volume and analyzed by paired *t* tests. *P<0.0001, †*P*<0.001, ‡*P*<0.01, nonsignificant (ns) *P*>0.05. All data are mean±95% confidence interval.

Cosinor analysis identified peak systolic blood pressure (SBP) at ≈ZT 18 hours, (Table S2) with the nadir at ≈ZT 6 hours. We therefore assessed the renal mRNA expression of genes associated with circadian control, corticosteroid action, and within the NCC regulatory cascade at these 2 time points (Figure 1A). Of the circadian genes, per2, cry 1, and bmal1 had a higher expression when animals were active; but there was no difference in expression of per1 or cry2 at these time points. The expression of the glucocorticoid-response genes, sgk1 and tsc22d3 (glucocorticoid-induced leucine zipper protein), was higher when mice were active than during the inactive phase. We found no day:night variation for the genes encoding the MR, the glucocorticoid receptor, or 11βHSD2. Co-localization of MR and glucocorticoid receptor with NCC was confirmed (Figure S4), but 11βHSD2 was only expressed in a small number of distal-DCT cells (Figure S5).

We found no day:night variation in *slc12a3* (encodes NCC) expression or in any other transcripts within the NCC regulatory cascade at these 2 time points. The transcriptional changes detected here are largely consistent with those published in the CIRCA database.^32^ We found no day:night variation for per1, cry2, and wnk1 expression. These genes do have a circadian transcriptional profile,^32^ but our time points of ZT6 and ZT18 correspond to midway point between the peak and nadir of expression.

At the protein level, total NCC abundance had no day:night variation (Figure 1B), whereas the abundance of NCC phosphorylated on Thr53 (henceforth pNCC) was higher during the active phase in both male (Figure 1C) and female mice (Figure S6). The phosphorylation of NCC on Ser71 had no day:night variation (Figure S7).

Phosphorylation of Thr53 increases the half-life of NCC in the apical membrane^33^ and closely correlates to transporter activity.^34^ We measured the excretion of NCC in urinary exosomes as a noninvasive surrogate of transporter abundance in the DCT apical membrane. As for pNCC abundance, exosomal NCC excretion had a marked day:night variation and was higher during the active phase (Figure 1D).

### Adrenalectomy Reduces NCC Expression and Blunts Day:Night pNCC Variation

**Figure 2.**
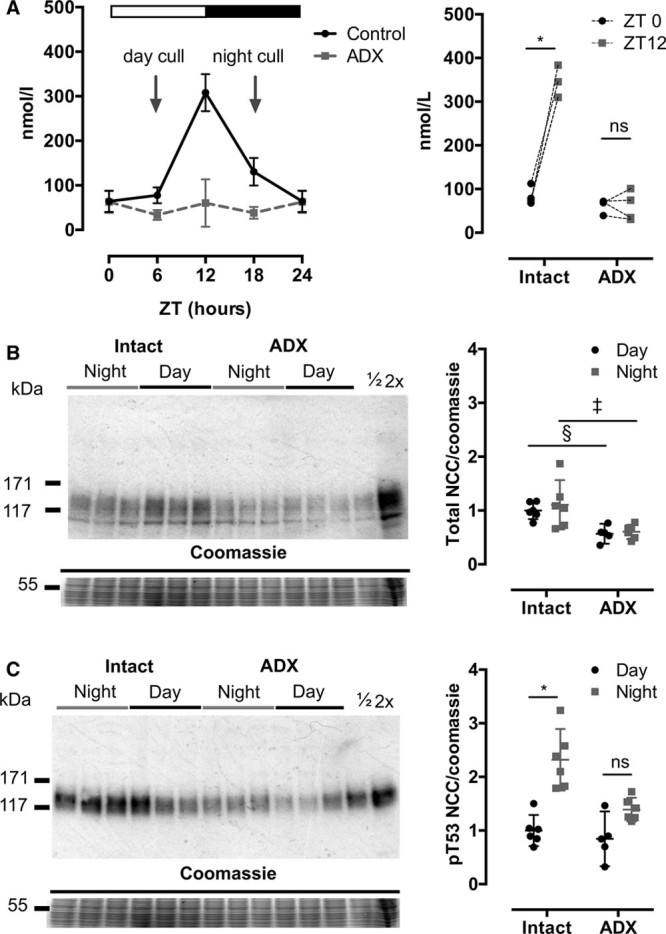
Day:night variation of plasma corticosterone (**A** and **B**), total NaCl cotransporter (NCC; **C**), and phosphorylated NCC (pNCC) protein (**D**) in adrenalectomized (ADX) and adrenal intact C57BL6 mice. A pooled profile of 24-hour plasma corticosterone was generated from composite terminal and spot plasma collections taken from ADX and control mice (**A**, n=8–20). Matched 2-way ANOVA was only performed on Zeitgeber time (ZT) 0 and ZT12 spot plasma samples collected concomitantly (**B**). The bar at the top of **A** and **B** indicates periods of light and dark. Kidneys were harvested at during the day when mice were asleep (ZT6) or at night when mice were awake (ZT18). Signal densities for immunoblots were normalized to Coomassie signal densities and compared with 2-way ANOVAs followed by post hoc Sidak correction. *P<0.0001, †*P*<0.001, ‡*P*<0.01, §*P*<0.05, nonsignificant (ns) *P*>0.05. All data are mean±95% confidence interval.

Adrenalectomy significantly reduced circulating corticosterone levels and abolished day:night variation (Figure 2A). Although slc12a3 mRNA levels were not changed by adrenalectomy (Figure S8), total NCC protein was reduced by ≈50% (Figure 2B). There was no significant day:night variation in pNCC levels in adrenalectomized mice (Figure 2C), and the variation in tsc22d3 expression was blunted (Figure S8). The day:night variation in the circadian genes per2, cry1, and bmal1 was maintained after adrenalectomy (Figure S8).

### Clamping Plasma Corticosterone Abolishes Daily pNCC Variation

**Figure 3.**
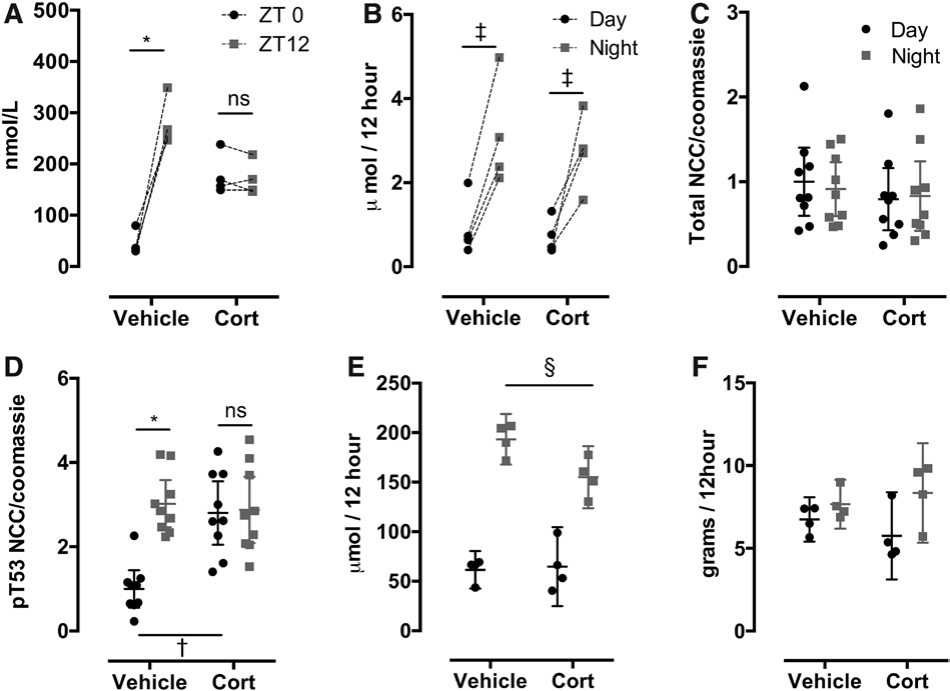
Day:night variation of plasma corticosterone (**A**), urinary aldosterone (**B**), total NaCl cotransporter (NCC; **C**), phosphorylated NCC (pNCC) protein (**D**), urinary sodium (**E**), and food intake (**F**) in chronic corticosterone (Cort)- and vehicle-treated C57BL6 mice. Plasma was sampled in Cort- and vehicle-treated mice at Zeitgeber time (ZT) 0 and ZT12. Urinary aldosterone and sodium were measured in samples collected during the 12 hours of subjective day, when mice were asleep (ZT0–12) and over subjective night when mice were awake (ZT12–0). Food intake was measured during this time. These urine and food intake data are the average for each mouse during 2 consecutive days’ measurements. Kidneys were harvested at ZT6 (day/sleep) and ZT18 (night/active) for immunoblots using whole kidney homogenate. Plasma corticosterone, urinary aldosterone and sodium excretion, and food intake data were compared with matched 2-way ANOVA with post hoc Sidak correction. Normalized signal densities for immunoblots were analyzed using 2-way ANOVA with post hoc Sidak correction. *P<0.0001, †*P*<0.001, ‡*P*<0.01, §*P*<0.05, nonsignificant (ns) *P*>0.05. All data are mean±95% confidence interval.

Next, we implanted slow-release corticosterone pellets, which eliminated the day:night variation and clamped corticosterone in the midphysiological range at ≈200 nmol/L (Figure 3A). The day:night variation in aldosterone levels was not affected by this maneuver (Figure 3B). Slc12a3 mRNA (Figure S9) and total NCC abundance (Figure 3C; Figure S10) were not changed by chronic corticosterone infusion but pNCC variation was abolished (Figure 3D; Figure S10). This reflected increased pNCC levels during the inactive phase in corticosterone-treated mice. Glucocorticoid clamping also increased expression of tsc2233 and sgk1 during the inactive period, as well as levels of cry1, bmal1 and per1 (Figure S9). A small but significant reduction in active phase urinary sodium excretion was recorded in corticosterone-treated mice (Figure 3E), which was not attributable to reduced food intake (Figure 3F). Sodium excretion during the inactive phase was not different from controls.

### Clamping Plasma Corticosterone Induces a Nondipping BP Profile

**Figure 4.**
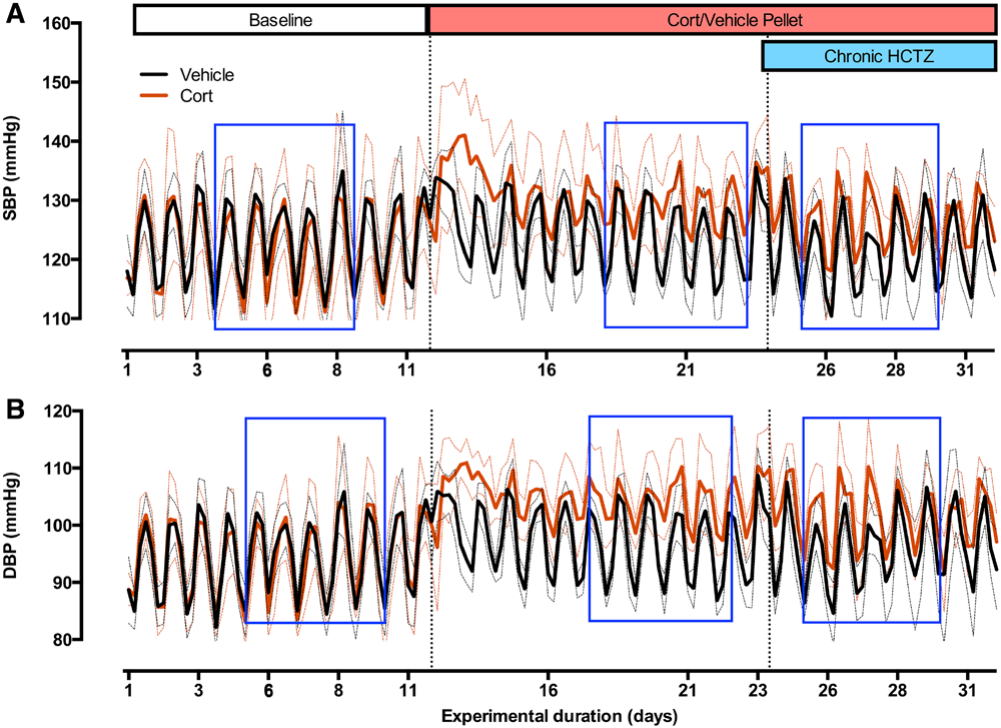
Systolic blood pressure (SBP; **A**) and diastolic blood pressure (DBP; **B**) in C57BL6 mice after treatment with corticosterone or vehicle followed by chronic hydrochlorothiazide (HCTZ). Moving averages (during 5 hours) throughout the duration of the experiment are displayed here. After baseline recordings (11 days), all mice were anesthetized under isoflurane and received either vehicle or corticosterone slow-release silastic pellets (subcutaneously). After 11 days, all mice were treated with chronic HCTZ (80 mg/kg) in their drinking water. The blue rectangles indicate 5-day bins that were taken forward for further analysis. All data are mean±95% confidence interval (CI), n=6 (vehicle, black line) n=5 (corticosterone, red line), where thick lines are the mean, and thin-dotted lines are 95% CI.

**Figure 5.**
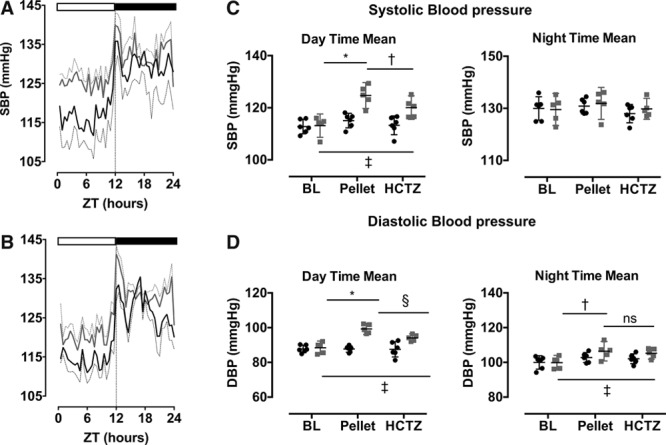
**A**, Corticosterone (gray lines) increased systolic blood pressure (SBP) when mice were asleep but not during the active period. **B**, Chronic hydrochlorothiazide (HCTZ) treatment lowered sleep-time SBP in corticosterone-treated mice (gray line) but not in controls (black line). The bar at the top of **A** and **B** indicates periods of subjective day (when mice were asleep) and night (when mice were awake). The analysis was performed on 5-day bins of raw SBP data (blue box in Figure 4) and a group average calculated every 30 minutes in the 24-hour cycle. The average SBP (**C**) and diastolic blood pressure (DBP; **D**) were also calculated during subjective day (Zeitgeber time [ZT] 3–8) when mice were asleep and night (ZT15–20), when mice were awake. Data were analyzed by matched 2-way ANOVA with post hoc Sidak correction. *P<0.0001, †*P*<0.001, ‡*P*<0.01, §*P*<0.05, nonsignificant (ns) P>0.05, n=6 (vehicle), n=5 (corticosterone). Data are mean±95% confidence interval (CI), where thick lines are mean and thin-dotted lines are 95% CI.

SBP (Figure 4A) and diastolic blood pressure (DBP; Figure 4B) were measured in mice by radiotelemetry before and after implantation of slow-release corticosterone (n=5) or control (n=6) pellets and then during thiazide therapy. Before implantation, daily rhythms of BP, heart rate, and locomotor activity were robust; acrophase, amplitude, and mesor were not different between groups (Table). Pellets were then implanted and, after recovery from anesthetic, the vehicle group immediately regained the daily rhythm of SBP (Figures 4A, 5A, and 5C) and DBP (Figures 4B and 5D), which were not different from preimplant values. The corticosterone-treated group took longer to regain 24-hour periodicity and when they did, SBP was raised during sleep compared with baseline values in all mice (Figure 5A and 5C). Corticosterone treatment increased DBP both in the active and in the inactive phases (Figure 5D). Changes to the day:night variation in SBP did not reflect changes in either locomotor activity or heart rate, as assessed by telemetry (Figure S11).

Cosinor analysis was performed on 5-day bins of steady-state SBP (indicated by the blue boxes on Figure 4A). Corticosterone treatment significantly (*P*<0.001) increased mesor and significantly decreased both the amplitude and robustness (*P*<0.001) of the underlying rhythm (Table).

### Chronic Thiazide Therapy Restores a Dipping BP Profile

In the next phase of the experiment, the mice were given hydrochlorothiazide in drinking water, achieving a plasma concentration of ≈15 μmol/L in both the groups. This did not alter BP in the control group but had a significant antihypertensive effect in the corticosterone-treated mice (Figure 5B). Thiazide reduced SBP during the inactive phase without affecting SBP during the active phase (Figure 5C). A similar profile was observed for DBP, being reduced by hydrochlorothiazide treatment when mice were asleep but remaining elevated during the active phase (Figure 5D). Cosinor analysis showed that hydrochlorothiazide restored a robust day:night rhythm for SBP in corticosterone-treated mice, reducing mesor and increasing amplitude to levels not significantly different from controls (Table).

## Discussion

We find that NCC has a day:night variation of activity determined by phosphorylation of existing protein rather than by gene transcription. This variation is influenced by the rhythm of circulating glucocorticoid. If this hormonal profile is flattened, NCC is inappropriately activated during the inactive phase, inducing a nondipping BP profile. Hydrochlorothiazide, which inhibits NCC, reduced BP during the inactive phase only in corticosterone-treated mice, restoring a robust day:night variation in BP.

### Daily NCC Activity Is Determined by Phosphorylation

Around 4000 genes in the DCT exhibit day:night variation at the transcriptional level, of which ≈10% fit a circadian profile with a period of 24 hours.^19^ Genes found with circadian rhythmicity included clock transcription factors, such as per1/2, cry1/2, and bmal1.^19,32^ Glucocorticoid-induced genes, such as the kinase sgk1 and the transcriptional regulator tsc22d3, also displayed a circadian profile of transcription at whole-kidney level.^32^ Our data are consistent with this literature (day:night transcriptional changes were found in per2, cry1, bmal1, sgk1, and tsc22d3); however, a limitation of our work is that it lacked the temporal resolution to detect rhythmic changes that have been reported for per1,^19,32^ *cry2*,^19,32^ *mo25α*,^32^ *cul3*,^32^ and WNK1^32^ expression. It is also important to note that we assessed transcription at the whole-kidney level and cannot, therefore, discriminate between glucocorticoid-sensitive regions of the kidney and those with minimal glucocorticoid sensitivity (ie, the aldosterone-sensitive distal nephron) because of expression of 11βHSD2. In such regions sgk1 and tsc22d3 might be regulated by aldosterone.

Slc12a3, which encodes NCC, had no day:night rhythm of transcription,^19,32^ as we have confirmed here. Our data at the whole-kidney level underscore the concept that day:night variation in NCC activity is regulated by post-transcriptional modification of existing protein.^19^ Of the 3 important residues thus far defined, our data show day:night control of phosphorylation of Thr53, but not Ser71. We do not present data for Thr58 phosphorylation, as we found significant cross-reactivity of this antibody with NKCC2, which is present in our samples.

pNCC abundance is often used as a surrogate marker for NCC activity as it correlates with thiazide-sensitive transport in cells^34,32^ and in the intact kidney.^29^ We chose not to assess thiazide-sensitive sodium transport directly because the requisite approaches either increase corticosterone^35^ or abolish day:night rhythms of renal function.^36^ We instead used the excretion of NCC in urine exosomes to provide a noninvasive snap-shot of transporter abundance in the apical membrane of the DCT.^37,38^ Exosomal NCC excretion was higher when mice were active, as recently described in healthy humans.^39^ In combination, these complementary experimental strategies indicate that thiazide-sensitive sodium transport is normally reduced during the inactive phase, correlating with the fall in BP and the lower ambient glucocorticoid level.

### How Do Glucocorticoids Influence Daily Variation in NCC Activity?

The circadian rhythm of circulating glucocorticoids can entrain renal clock gene transcription.^22^ One hypothesis is that circadian transcription factors are the link between circulating corticosterone and phosphorylation/activation of NCC. Per1 does regulate NCC activity, binding the promotor of *slc12a3* and *wnk4* to positively regulate transcription in a DCT cell line.^20^ In our study, adrenalectomy abolished the daily variation of pNCC abundance but did not uniformly affect the variation in clock gene expression. A clearer relationship was observed in mice chronically infused with glucocorticoid. In this scenario, increased pNCC levels during the inactive phase were associated with increased transcription of per1, cry1, and bmal1. A clearer relationship to pNCC abundance across all experimental paradigms was with sgk1 and tsc22d3 expression. An alternative hypothesis is that the circadian variation in glucocorticoids directly controls NCC activity by engaging a network of regulatory kinases downstream of a corticosteroid receptor and upstream of the WNK/SPAK/OSR1 kinase cascade. Indeed, synthetic glucocorticoids increase the expression/activity of NCC,^40,41^ and recent studies show that sgk1 enhances NCC activity indirectly by suppressing degradation of WNK1 kinase.^42^ Because we found that most DCT cells do not express 11βHSD2, corticosterone could induce sgk1 expression through either MR or glucocorticoid receptor activation^43^: MR is the most plausible route because MR antagonists abolish the endogenous rhythm of the WNK1/SPAK/OSR1 phosphorylation cascade.^19^ Nevertheless, an important limitation to our study is that gene expression and protein phosphorylation were assessed at only 2 time points. This lack of temporal resolution precludes cosinor analysis of these data sets and limits our ability to assess the relationship between glucocorticoid-activated transcriptional pathways and NCC activation.

### How Do Glucocorticoids Induce a Nondipping BP Profile?

We chronically infused corticosterone such that plasma levels were held midway between the physiological peak and trough. This rapidly induced nondipping BP, reflecting a significant rise in BP during the inactive phase. Our approach models the loss of nocturnal BP observed in patients receiving chronic glucocorticoid therapy.^25^ Glucocorticoid insufficiency in humans and adrenalectomy in mice also achieve a flat daily cortisol/corticosterone profile. In this setting, the reduced day:night BP variation reflects a low-peak BP during waking hours.^26,35^ Overall, these data indicate that an intact glucocorticoid rhythm is essential for the normal daily variation in BP: loss of this dynamic glucocorticoid control may contribute to increased cardiovascular mortality in patients on chronic glucocorticoid therapy.^36^

What accounts for the loss of dip in our study? It is unlikely to reflect increased sympathetic drive in the inactive period,^37^ since the daily variation in heart rate was not affected by corticosterone treatment. We also discount a major role for reduced arterial compliance^38^ because DBP increased (throughout the 24-hour cycle), rather than fell, during corticosterone treatment. The most plausible explanation of our data are that corticosterone activates the WNK/SPAK/OSR1 cascade and clamps NCC on throughout the 24-hour cycle. This impairs the normal modulation of BP by pressure natriuresis, and in consequence BP does not fall during sleep to facilitate sodium excretion and maintain balance.^39^ Indeed, corticosterone infusion did not affect overall sodium balance but reduced sodium excretion during the active phase. This is consistent with data from normotensive humans: those excreting a greater proportion of the daily sodium load during sleep have a reduced dip during sleep.^12^ This concept is reinforced by data in adrenalectomized mice. These animals do not activate NCC during periods of activity and cannot maintain sodium balance by modulating renal excretion: BP lacks day:night variation because peak BP is significantly reduced.

### Perspectives

Hydrochlorothiazide monotherapy was previously shown to reduce night-time BP in essential hypertensive patients, but only those with a nondipping BP profile.^15^ Night-time dosing of hydrochlorothiazide (in combination with valsartan) is more effective than daytime dosing at reducing BP during sleep.^17^ Our study provides mechanistic insight into these outcomes and suggests that thiazides, or thiazide-like diuretics with a longer half-life could improve BP control over the 24-hour cycle in certain patients. Measuring urinary exosomal NCC in morning urine samples would be an effective and noninvasive means of patient stratification. We predict benefit in nondipping patients on glucocorticoid therapy or those with abnormal peripheral glucocorticoid metabolism. Restoring dipping BP profile through thiazide diuretics would be a cost-effective way of reducing cardiovascular risk in these patients.

## Acknowledgments

T.S. Peltz was awarded a scholarship by The Physiological Society. We thank the British Heart Foundation Centre of Research Excellence Award for supporting the telemetry studies.

## Sources of Funding

J.R. Ivy and A.R. Howarth were funded by PhD studentships from The British Heart Foundation (FS/11/78/29328 and FS/13/52/30637).

## Disclosures

None.

## Supplementary Material

## Novelty and Significance

### What Is New?

- Persistent elevation of circulating glucocorticoids induced a nondipping blood pressure (BP) profile.
- This nondipping BP was associated with persistent activation of the renal sodium chloride cotransporter.
- Treatment with a thiazide diuretic reduced BP and restored the normal dipping rhythm.

### What Is Relevant?

- BP normally dips during sleep and loss of the nocturnal dip (nocturnal hypertension) increases the risk of cardiovascular and renal disease.
- Elevated glucocorticoid levels may promote the nocturnal hypertension common in chronic stress, obesity, and type 2 diabetes mellitus.
- Long-lasting thiazide diuretics may improve BP control throughout the 24-hour cycle.

### Summary

This study suggests that thiazide diuretics may lower nocturnal blood pressure in conditions associated with abnormal glucocorticoid metabolism, reducing long-term cardiovascular risk in such patients.
