# Supplementary material for: Glucocorticoids Induce Nondipping Blood Pressure by Activating the Thiazide-Sensitive Cotransporter
Source: Hypertension. 2016 Apr 13;67(5):1029–37. doi: 10.1161/HYPERTENSIONAHA.115.06977 (PMC4905621; doi:10.1161/HYPERTENSIONAHA.115.06977)
Supplement: Supplementary file 1 [file hyp-67-1029-s001.docx]

**SUPPLEMENTARY MATERIAL**

**Glucocorticoids induce non-dipping blood pressure by activating the thiazide-sensitive co-transporter**

**Jessica R. Ivy^1^, Wilna Oosthuyzen^1^, Theresa Peltz^1^, Amelia R. Howarth^1^, Robert W. Hunter^1^, Neeraj Dhaun^1^, Emad A.S. Al-Dujaili^2^, David J. Webb^1^, James, W. Dear^1^, Peter W. Flatman^3^, Matthew A. Bailey^1^**

^1^The British Heart Foundation Centre for Cardiovascular Science The University of Edinburgh; ^2^Dietetics, Nutrition and Biological Sciences Department, Queen Margaret University and ^3^The Centre for Integrative Physiology, The University of Edinburgh, United Kingdom

Correspondence to: Matthew A. Bailey, PhD

University/BHF Centre for Cardiovascular Science,

The Queen’s Medical Research Centre,

The University of Edinburgh,

47 Little France Crescent,

Edinburgh, EH16 4TJ United Kingdom

Email [Matthew.Bailey@ed.ac.uk](mailto:Matthew.Bailey@ed.ac.uk)

**Supplementary Methods:**

***Measurement of NCC in urinary exosomes***: The number of exosomes in urine samples was measured by Nanoparticle Tracking Analysis (NTA, NanoSight Ltd, Amesbury, UK), as described^1^. NCC-containing exosomes were identified by fluorescence after conjugation of a rabbit anti-NCC antibody (AB3553; EMD Millipore) to a quantum dot (Qdot 605 Anti- body Conjugation Kit; Invitrogen, UK). For fluorescent NTA analysis, a 532nm (green) laser diode excited the Qdots with a long-pass filter (430nm) so that only fluorescent particles were tracked and labeled-particle concentration determined by NTA software.

***Immunoblotting***: Western blots were performed as we have described^2^. Briefly, kidneys were homogenized in a sucrose buffer containing phosphatase, kinase, and protease inhibitors, and protein samples were resolved by SDS-PAGE (NuPAGE Novex 3%–8% Tris-acetate gels; Invitrogen, UK) and transferred to PDVF membrane (Amersham Hybond-P GE Healthcare). Primary antibodies used were: rabbit anti-NCC (AB3553; EMD Millipore, 1:1000); sheep anti-pThr53-NCC, anti-pThr58-NCC, anti-pSer71-NCC (each at 1:500), and anti-NKCC2 (1:10,000, all from Division of Signal Transduction Therapy, Dundee University, UK). Secondary antibodies were: goat anti-rabbit IgG-HRP (sc-2030; Santa-Cruz Biotechnology, 1:2000), and donkey anti-sheep IgG-HRP (A3415; Sigma-Aldrich, 1:20,000). Images were developed by electrochemiluminescence and quantified by densitometry using ImageJ. The bottom <50kDa of the gel was coomassie stained and used as a loading control, as described^3^. The cross-reactivity of NCC phospho-antibodies was determined by immunoprecipitation experiments. NCC and NKCC2 were separately pulled-down from whole kidney homogenates and the products probed using pThr53-NCC, pThr58-NCC and pSer71-NCC antibodies. Anti-pThr53-NCC and anti-pSer71-NCC were selective for NCC but anti-pThr58-NCC showed significant cross-reactivity with NKCC2 (See Supplemental Material, Figure S1).

***Quantitative-PCR***: RNA was extracted from frozen kidneys (RNeasy Mini Kit, Qiagen) and treated on-column with DNase I. cDNA was transcribed from 500 ng of total RNA and used for qPCR reactions with the Roche Universal ProbeLibrary (See Supplemental Material, Table 1). TBP, HPRT and 18S rRNAs were used as endogenous control genes. Their expression did not differ across any of the experimental groups. CT values were approximately 25-33, 22-28, 7-15 for TBP, HPRT and 18S, respectively. Test gene CT values ranged from 22 to 37. The level of endogenous control genes was normalized such that day/vehicle/sham was equal to 1.

## Immunofluorescence: Kidneys were fixed by aortic perfusion of 4% paraformaldehyde and then embedded in paraffin, sectioned at 5μm and mounted onto glass slides. Sections were double-immunostained to co-localise NCC expression with GR, MR or 11βHSD2; all sections were counterstained with DAPI (Immunodetection and Imaging Facility; The University of Edinburgh). Images were acquired using a Zeiss LSM 510 Meta Confocal Laser Scanning Microscope. DAPI, Cy3 and Cy5 were excited using a blue diode 405nm laser and HeNe 546nm and 633nm lasers respectively. Tiled images (~7x7) were captured through a 20X objective lens.

***BP measurement*:** Radiotelemetry devices (Model TA-11PAC-10, Data Systems International, UK) were inserted into male C57BL/6 mice under isoflurane anesthesia. After a week of post-operative recovery, data were collected over a 1-minute period every 30 minutes at an acquisition rate of 1kHz. Basal measurements were obtained over 7 days before corticosterone (n=5) or blank (n=6) elastomer pellets were implanted subcutaneously. In order to verify that the plasma corticosterone clamping was sustained throughout the experiment, blood was sampled at ZT0 and ZT12 in vehicle and corticosterone treated mice on the last day of the telemetry experiment i.e. four weeks after implantation. After 11 days, hydrochlorothiazide (HCTZ; 80mg/kg/day) was administered in drinking water. The mice did not change their intake of water following HCTZ dosing. HCTZ concentration in plasma samples taken on the final experimental day was measured by LC/MS.

**REFERENCES**

1. Oosthuyzen W, Sime NE, Ivy JR, Turtle EJ, Street JM, Pound J, Bath LE, Webb DJ, Gregory CD, Bailey MA, Dear JW. Quantification of human urinary exosomes by nanoparticle tracking analysis. *J Physiol*. 2013;591:5833-5842.

2. Hunter RW, Ivy JR, Flatman PW, Kenyon CJ, Craigie E, Mullins LJ, Bailey MA, Mullins JJ. Hypertrophy in the Distal Convoluted Tubule of an 11beta-Hydroxysteroid Dehydrogenase Type 2 Knockout Model. *J Am Soc Nephrol*. 2015;26:1537-1548.

3. McDonough AA, Veiras LC, Minas JN, Ralph DL. Considerations when quantitating protein abundance by immunoblot. *Am J Physiol Cell Physiol*. 2015; 308:C426-C433.

Table S1: Table of qRT-PCR assays.

| **gene** | **accession number** | **forward primer** | **reverse primer** | **UPL** |
| --- | --- | --- | --- | --- |
| *Rn18s* | NR_003278.1 | ctcaacacgggaaacctcac | cgctccaccaactaagaacg | 77 |
| *Tbp* | NM_013684 | gggagaatcatggaccagaa | gatgggaattccaggagtca | 97 |
| *HPRT* | NM_013556.2 | cctcctcagaccgcttttt | aacctggttcatcatcgctaa | 95 |
| *per1* | NM_001159367.1 | gcttcgtggacttgacacct | tgctttagatcggcagtggt | 71 |
| *per2* | NM_011066 | gcttcgtggacttgacacct | tgctttagatcggcagtggt | 5 |
| *clock-201* | NM_007715.5 | ccagtcagttggtccatcatt | tggctcctaactgagctgaaa | 76 |
| *cry1* | NM_007771.3 | ggcagagcagtaactgatacga | tgactttcccaccaacttca | 52 |
| *cry2* | NM_009963.4 | ggagcatcagcaacacagg | ccgcttggtcagttcttcac | 11 |
| *arnt1 var1* | NM_001243048 | gaatacattgtctcaaccaacactg | ttagctgcgggaaggttg | 97 |
| *arnt1 var2* | ENSMUST00000106637.1 | agtacgcctccccctgat | tgtctggagtccctccattt | 79 |
| *Sgk1* | NM_001161845 | gattgccagcaacacctatg | ttgatttgttgagagggacttg | 91 |
| *TSC22d3 v2* | NM_010286.3 | tccgttaaactggataacagtgc | tggttcttcacgaggtccat | 49 |
| *MR* | NM_001083906 | caaaagagccgtggaagg | tttctccgaatcttatcaataatgc | 11 |
| *GR* | NM_008173 | tgacgtgtggaagctgtaaagt | catttcttccagcacaaaggt | 56 |
| *Hsd11b2* | NM_008289.2 | cactcgaggggacgtattgt | gcaggggtatggcatgtct | 26 |
| *Slc12a3* | *NM_019415* | *cctccatcaccaactcacct* | *ccgcccacttgctgtagta* | 12 |
| *Oxsr1* | NM_133985 | tgccttcaaaaggatccaga | tggaaaaatttgtgcctcaac | 84 |
| *Stk39* | ENSMUST00000102715 | gtacgagctccaggaggttatc | tcttgcctgggtttgcat | 27 |
| *Stk39* | JN368425 | ttaccgtcattcctaactttactgc | gaatgcgcttactccaaaatct | 18 |
| *Wnk1* | AY309076 | cttttgccaagagtgtgataggt | caacggattcatcatatttctcc | 92 |
| *Wnk1* | AY311934 / AY309076 | tgctgctgttctcaaaagga | acttcaggaattgctactttgtca | 20 |
| *Wnk4* | NM_175638 | tccgatttgatctggatgg | gggcaggatgaactcattgta | 26 |
| *Nedd4* | NM_010890 | acgtgctgttcactgctgat | tcacaactcgtgtgtcatcg | 1 |
| *Cab39* | NM_133781 | tgagaaggagcctcagacaga | aaaatttgagccacgtcttttt | 17 |
| *Cul3* | NM_016716.4 | agaagggaagaatcctgttgact | gccggtcattattgaaggatt | 69 |

Table S2: Parameters obtained from cosinor analysis of C57BL6 mice telemetry data. Data are mean± 95% CI. (n=4)

| **Variable** | **SBP** | **DBP** | **HR** | **Activity** |
| --- | --- | --- | --- | --- |
| **Period (hours)** | 23h09 ±0h22 | 23h09 ±0h18 | 23h48 ± 0h12 | 23.48 ±0h12 |
| **Acrophase (ZT)** | 18h10±0h47 | 18h09±0h49 | 17h06±0h05 | 17h06±0h05 |
| **Amplitude (mmHg/bpm/counts)** | 10.0 ± 1.8 | 9.8 ± 1.5 | 57.0 ± 22 | 6.24 ± 1.2 |
| **MESOR (mmHg/bpm/counts)** | 125 ± 2 | 95 ± 5 | 507 ± 7 | 7± 2 |
| **Robustness (%)** | 26.3±4.3 | 23.9±3.3 | 12.5±5.0 | 28.5±1.9 |

***Immunoprecipitation (IP) for specificity of NCC phospho-antibodies:***

The NCC phospho-motifs are conserved in other related transporters, particularly NKCC2. Threonine 53 and 58 on NCC shares homology with Threonine 96 and 101 on NKCC2. Therefore IP was performed to test the specificity of the NCC phospho-antibodies. Superparamagnetic beads (Dynabeads^TM^ Protein G, Life Technologies) were washed and conjugated to NCC and NKCC2 antibodies according to the manufacturer’s instructions. 2500mg kidney homogenate was added to the bead-antibody mixture and placed on a magnet to “pull-down” proteins bound to the antibodies. The antigens were eluted and resuspended (in 20μL Dynabeads^TM^ elution buffer, Life Technologies, 3μL DTT and 7.5μL LDS) and, along with a standard kidney homogenate, were Western blotted.


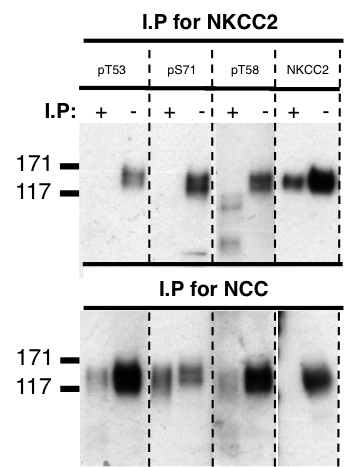


Figure S1: Cross-reactivity of anti-NCC antibodies

Immunoprecipitation was used to “pull down” NCC or NKCC2 from 2500 μg of total kidney homogenate. These immunoprecipitation products (+) were then separated on 3-8% Tris Acetate gel, transferred to Hybond P membrane and immunoblotted for the NCC phospho-antibodies and NKCC2. 12 μg of total kidney homogenate (-) was run alongside the IP products and probed directly as a positive control.

Figure S2: 24-hour systolic blood pressure (A), diastolic blood pressure (B), heart rate (C) and activity (D) in C57BL6 mice. Data were collected using radiotelemetry and are shown as hourly mean over 5-day batches (thick line) ±95% CI (thin dotted line) (n=4). The white bar indicates subjective day, when mice were inactive; the black bar indicates the subjective night, when mice were active. Lights were turned on at 7am local time (ZT0).

Figure S3: Urinary aldosterone (A), corticosterone (B), sodium (C) and potassium (D) excretion and food intake (E). Urine samples were collected over subjective day (black circle; ZT0-12), when mice were inactive and over the night (red square; ZT12-0), when mice were active. These data are the average for each mouse over two consecutive days’ measurements. Data are mean ±95% CI. Statistical comparisons were made using paired Student’s *t* tests, ****P<0.0001 ***P<0.001. **P<0.01 (n=10).


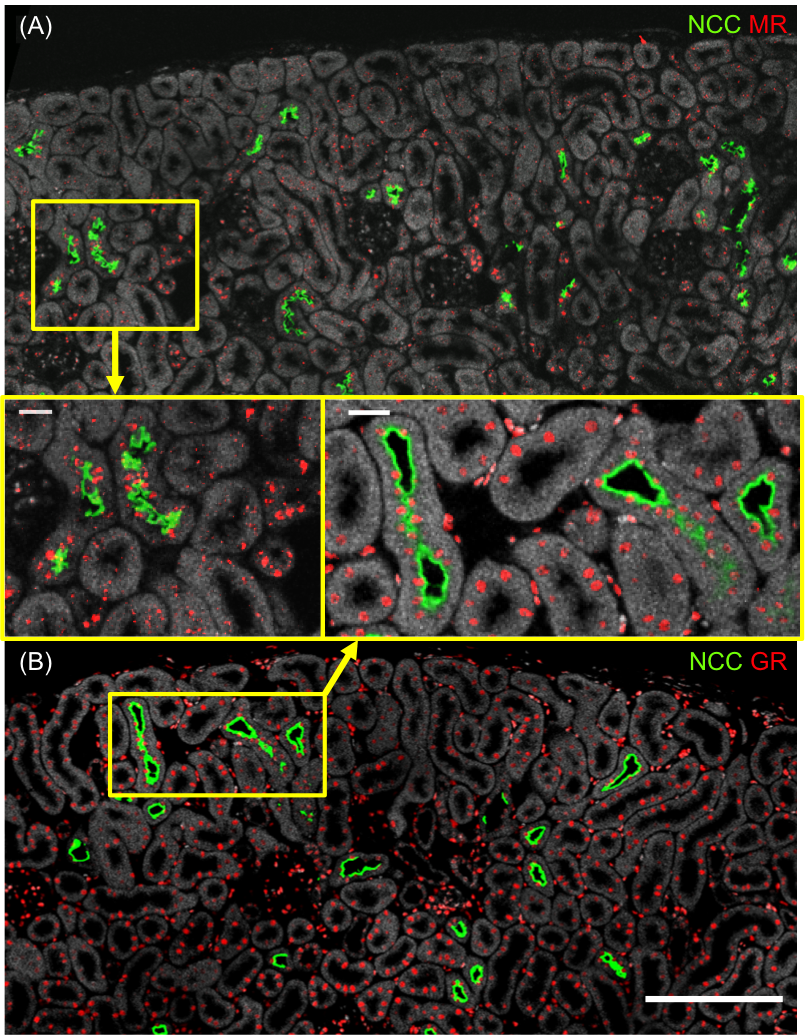


Figure S4: Representative images of immunofluorescent staining of (A) NCC (green) and MR (red) and (B) NCC (green) and GR (red) in male C57BL6 mice. High-powered views of yellow-boxed regions are displayed. Bar, 100μm in large top/bottom panels; 10μm in high-powered middle panels.


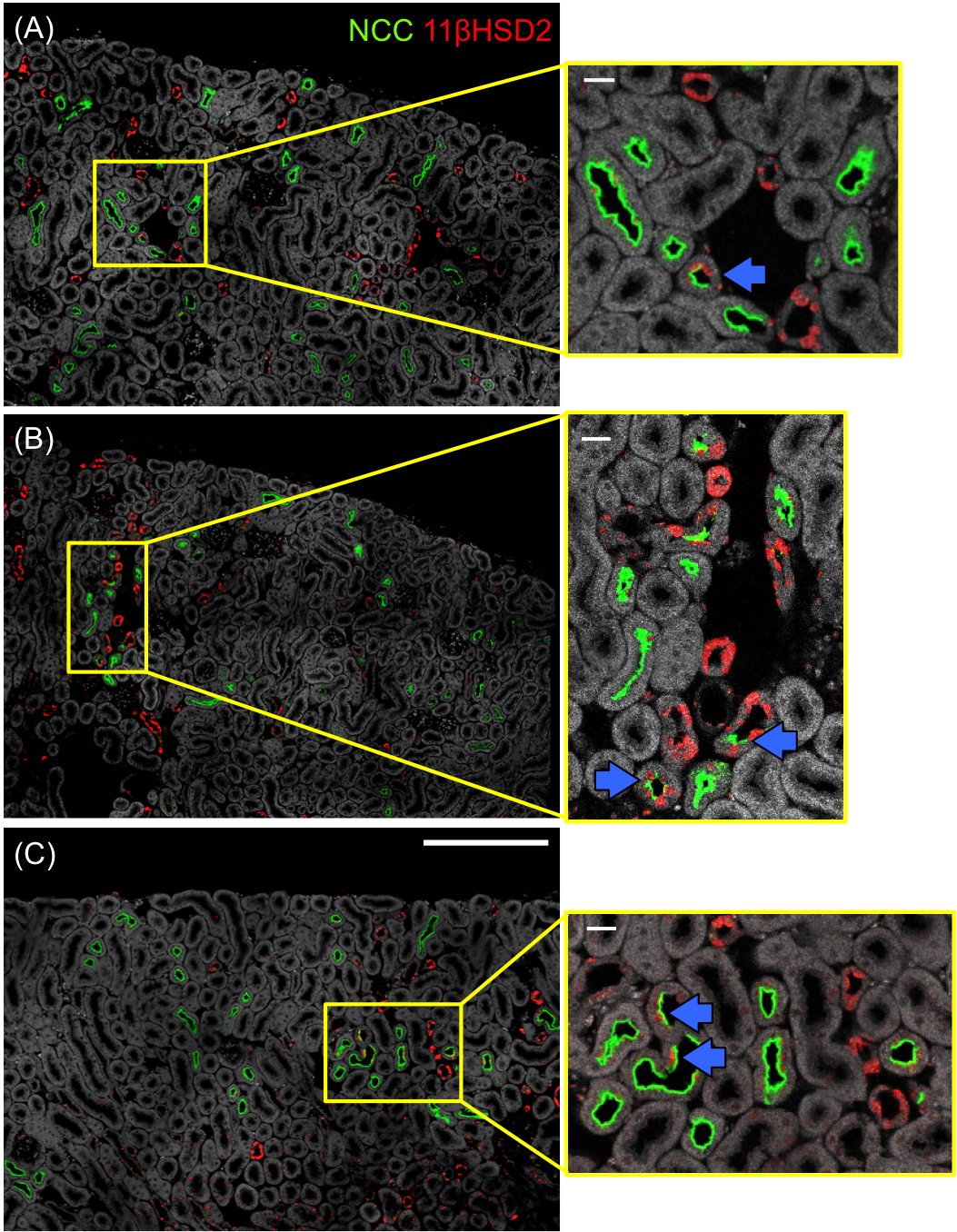


Figure S5: Immunofluorescent staining for NCC (green) and 11HSD2 (red) in 3 (A-C) male C57BL6 mice. High-powered views of yellow-boxed regions are given on the right where blue arrows indicate regions in which 11βHSD2 and NCC staining are within the same cell/tubule. Bar, 100μm (left-hand panels); 10μm (right hand panels).

Figure S6: Day:night variation of NCC phosphorylation at Thr53 in female C57BL6 mice. Kidneys were harvested at ZT6 (day/inactive phase) and ZT18 (night/active phase) for immunoblots. No attempt was made to synchronize the oestrus cycles of these mice. Signal densities for immunoblots were normalised to Coomassie signal density and compared by unpaired *t*-tests, **P<0.01, All data are mean ± 95% CI.

Figure S7: Day:night variation of NCC phosphorylation at Ser71 in male C57BL6 mice. Kidneys were harvested at ZT6 (day/inactive phase) and ZT18 (night/active phase) for immunoblots. Signal densities for immunoblots were normalised to Coomassie signal density and compared by unpaired *t*-tests. ns, P>0.05. All data are mean ± 95% CI

Figure S8: Gene expression in kidneys of adrenalectomised (ADX) and adrenal intact C57BL6 mice. Control and ADX mice were single housed and culled at day 9 post surgery during the subjective day when inactive (black circle; ZT6) or night (red square; ZT18), when active. The encoding gene is given with name of the resulting protein in brackets. Gene expression of each gene was normalised to the average expression of 18S, TBP and HPRT. Data are mean ± 95% CI. Data were analysed with two-way ANOVA followed by *post hoc* Sidak correction, where ***P<0.001, **P<0.01 *P<0.05, ns P>0.05 (n=5-6).

******

Figure S9: Gene expression in kidneys of corticosterone (Cort) or vehicle treated C57BL6 mice. Mice were culled during the subjective day when inactive (black circle; ZT6) or night (red square; ZT18), when active.

The encoded protein name is given in brackets. Gene expression of each test gene was normalised to the average expression of 18S, TBP and HPRT. Data are mean ± 95% CI. Data were analysed with two-way ANOVA followed by *post hoc* Sidak correction, where ****P<0.0001, ***P<0.001, **P<0.01 *P<0.05, ns P>0.05 (n=9) *
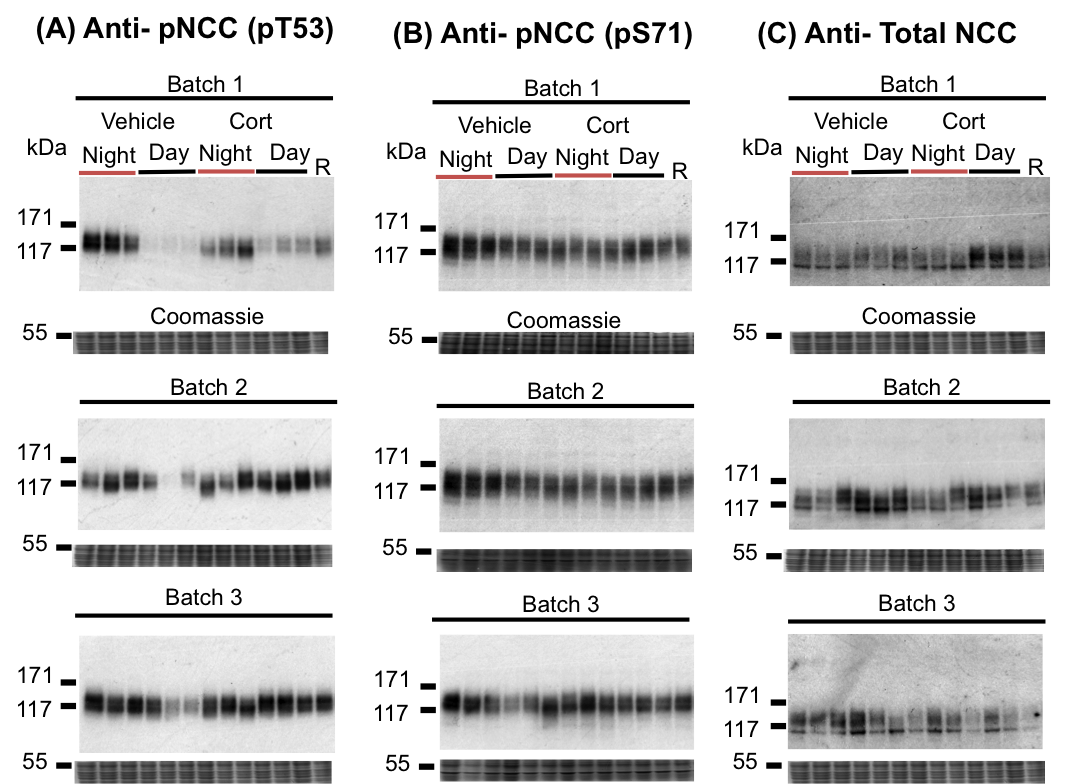
*

Figure S10: Immunoblots for pT53 NCC (A) pS71 NCC (B) and total NCC (C) in vehicle and corticosterone treated mice. Mice were culled in groups of 3 control and 3 experimental animals during the subjective day when inactive (ZT6) or night (ZT18), when active. The experiment was performed on three separate occasions and immunoblots were performed in these batches (1-3). “R” is reference load, which was a pooled sample that was used to normalise across the three batches of immunoblots.

Figure S11: Heart rate and activity in C57BL6 mice. After 11 days of baseline recording, mice received either corticosterone (red line) or control (black line) pellets. After a further 11 days, all mice received HCTZ (80 mg/kg) in their drinking water. Data displayed here are the moving average (over 5 hours) of heart rate (A) and activity (D) throughout the duration of the experiment. The blue rectangles indicate 5-day bins that were taken forward for further analysis. Summary mean daytime and nighttime heart rate (B, C) and activity (E, F) were obtained by taking the mean over the 5-day bins for subjective day, when mice were inactive (ZT3-8) and night (ZT15-20), when mice were active. Data are mean ± 95% CI, where thick lines are mean and feint lines are 95% CI. Summary data were analysed by matched two-way ANOVA with *post hoc* Sidak correction. ****P<0.0001, ***P<0.001, **P<0.01, *P<0.05 ns, not significant P>0.05, n=6 (vehicle) n=5 (corticosterone).
